# Dynamics and mechanisms of quantum dot nanoparticle cellular uptake

**DOI:** 10.1186/1477-3155-8-13

**Published:** 2010-06-15

**Authors:** Yan Xiao, Samuel P Forry, Xiugong Gao, R David Holbrook, William G Telford, Alessandro Tona

**Affiliations:** 1 Chemical Science and Technology Laboratory, National Institute of Standards and Technology (NIST), Gaithersburg, MD, USA; 2 Research and Development, Translabion, Clarksburg, MD, USA; 3 Experimental Transplantation and Immunology Branch, Center for Cancer Research, National Cancer Institute, National Institutes of Health, Bethesda, MD, USA; 4 Science Applications International Corporation (SAIC), Arlington, VA, USA

## Abstract

**Background:**

The rapid growth of the nanotechnology industry and the wide application of various nanomaterials have raised concerns over their impact on the environment and human health. Yet little is known about the mechanism of cellular uptake and cytotoxicity of nanoparticles. An array of nanomaterials has recently been introduced into cancer research promising for remarkable improvements in diagnosis and treatment of the disease. Among them, quantum dots (QDs) distinguish themselves in offering many intrinsic photophysical properties that are desirable for targeted imaging and drug delivery.

**Results:**

We explored the kinetics and mechanism of cellular uptake of QDs with different surface coatings in two human mammary cells. Using fluorescence microscopy and laser scanning cytometry (LSC), we found that both MCF-7 and MCF-10A cells internalized large amount of QD655-COOH, but the percentage of endocytosing cells is slightly higher in MCF-7 cell line than in MCF-10A cell line. Live cell fluorescent imaging showed that QD cellular uptake increases with time over 40 h of incubation. Staining cells with dyes specific to various intracellular organelles indicated that QDs were localized in lysosomes. Transmission electron microscopy (TEM) images suggested a potential pathway for QD cellular uptake mechanism involving three major stages: endocytosis, sequestration in early endosomes, and translocation to later endosomes or lysosomes. No cytotoxicity was observed in cells incubated with 0.8 nM of QDs for a period of 72 h.

**Conclusions:**

The findings presented here provide information on the mechanism of QD endocytosis that could be exploited to reduce non-specific targeting, thereby improving specific targeting of QDs in cancer diagnosis and treatment applications. These findings are also important in understanding the cytotoxicity of nanomaterials and in emphasizing the importance of strict environmental control of nanoparticles.

## Background

The arsenal of nanomaterials keeps expanding over the years as a result of the rapid growth of the nanotechnology industry. Nanomaterials are currently being used in a number of applications, including textiles, cleaning products, sport equipments, biomedicine, and cosmetics [1]. While the potential benefits of nanotechnology have been widely reported, little is known about the potential toxicity of nanomaterials [2]. The increasing use of nanoparticles in consumer products and medical applications underlies the importance of understanding any toxic effects to humans and the environment that have raised concerns over the years.

Among various nanomaterials, quantum dots (QDs) distinguish themselves in their far-reaching possibilities in many avenues of biomedicine. QDs are nanometer-sized fluorescent semiconductor crystals with unique photochemical and photophysical properties. Their much greater brightness, rock-solid photostability and unique capabilities for multiplexing, combined with their intrinsic symmetric and narrow emission bands, have made them far better substitutes for organic dyes in existing diagnostic assays [3]. These properties, combined with the development of ways to solubilize QDs in solution and to conjugate them with biological molecules, have led to an explosive growth in their biomedical applications [4]. Bioconjugated QD fluorescent probes offer a promising and powerful imaging tool for cancer detection, diagnosis and treatment. Following the two seminal papers published on *Science *in 1998 demonstrating the feasibility of using QDs in biological environments [5,6], many new techniques have been developed during the last decade, utilizing the unique photophysical properties of QDs, for *in vitro *biomolecular profiling of cancer biomarkers, *in vivo *tumor imaging, and dual-functionality tumor-targeted imaging and drug delivery [7].

Early detection of cancer and targeted drug delivery remain the primary challenges to the cancer research community. In many cases, the malignancy of tumors is detected only at advanced stages when high dose of chemotherapeutic drugs are needed, which raises the cost of the therapy as well as the risk of side-effects. To mitigate this problem, early detection of tumors at their incipient stage and targeted drug delivery system 'pinpointing' cancer cells at the tumor site is the key. A tumor-targeting drug delivery system generally consists of a tumor-recognition moiety and a drug-loaded vesicle. Currently, most drugs are designed to bind to specific receptors. However, these drugs lack selectivity for specific sites in the human body, *i.e*., specific cells, tissues or organs, since the receptors may be expressed at various sites of the body. Nanoparticles for site-specific drug delivery represent a promising solution to this problem. Mediated by a targeting sequence, drug-laden nanoparticles should deliver their payload only to specific target cells, tissues or organs under ideal circumstances [8]. A premise of nanomedicine is that it may be feasible to develop multifunctional constructs combining diagnostic and therapeutic capabilities, thus leading to better targeting of drugs to diseased cells. The large surface area combined with versatile surface chemistry makes QDs convenient scaffolds to accommodate anticancer drugs either through chemical linkage or by simple physical immobilization, leading to the development of nanostructures with integrated imaging and therapy functionalities [7]. Such a system is capable of targeting drug delivery and imaging the delivery process simultaneously to monitor the time course of subcellular location. Several studies have appeared recently highlighting this application [9-12].

In such applications as cancer diagnosis and drug delivery, specific uptake of QDs by cancer cells is desired while non-specific uptake by any cell type should be avoided. Otherwise, specific targeting of cancer cells cannot be achieved, as every cell, even the healthy ones, would be targeted. In this regard, understanding the mechanism of QD cellular uptake and factors affecting the process is essential to minimize unwanted non-specific cellular uptake of QDs. Unfortunately, the endocytic mechanism of non-targeting QDs (i.e., not bearing special functionalization targeting specific component of the cell) has been poorly studied and remains largely unknown, with only a few studies appeared recently to addressed this question [13-15]. In the present study, we used fluorescence microscopy, laser scanning cytometry (LSC), live cell fluorescent imaging and transmission electron microscopy (TEM) to explore the kinetics and mechanism of cellular uptake of QDs with different surface coatings by two different cell types representing normal and cancerous cells. In addition, the localization of QDs in the cytoplasm was examined with specific organelle markers. The findings presented here provide information on the mechanism of QD endocytosis that could be exploited to reduce non-specific targeting, thereby improving specific targeting of QDs in cancer diagnosis and treatment applications. These findings are also important in understanding the cytotoxicity of nanomaterials in general and in emphasizing the importance of strict environmental control of nanoparticles.

## Methods

### Quantum dots

QDs with emission maxima at 655 nm (QD655) were obtained from Invitrogen (Carlsbad, CA). These QDs have a CdSe core and a ZnS shell with three different surface coatings: carboxylic acids (COOH), amine-derivatized PEG, or PEG only, which were sold under the names Qdot 655 ITK carboxyl (Cat. No. Q21321MP), Qdot 655 ITK amino (PEG) (Cat. No. Q21521MP), and Qtracker 655 non-targeted (Cat. No. Q21021MP) quantum dots, respectively. At physiological pH, the surface charges on these coatings are negative, positive, or neutral, respectively.

### Cell culture

Human mammary non-tumorigenic epithelial cell line MCF-10A and human mammary adenocarcinoma epithelial cell line MCF-7 were obtained from ATCC (Manassas, VA) and cultured under conditions as recommended by the supplier.

### QDs cellular uptake

Cells were grown on tissue culture chamber slides (Nunc, Rochester, NY) to a density of 30,000 cells/cm^2^, and then incubated with QD655-COOH, QD655-amine PEG and QD655-PEG at 37°C for 12 h at final concentrations of 0.8, 0.5, and 0.8 nM respectively. Afterwards, cells were washed 3 times with PBS and then fixed in 10% neutral-buffered zinc formalin (Fisher, Pittsburgh, PA) for 45 min. Afterwards, the cells were counterstained with 4',6-diamidino-2-phenylindole-2 (DAPI) from Vector Laboratories (Burlingame, CA) and viewed directly under fluorescence microscopy or analyzed by laser scanning cytometry (see next section) as described previously [16-18].

### Laser scanning cytometry (LSC)

Samples were analyzed on a LSC2 laser scanning cytometer of Compucyte Corporation (Cambridge, MA) equipped with 405, 488, and 594 nm lasers and four PMT detectors. QD655 was excited with a violet laser diode (405 nm, 15 mW) and detected through 660/20 bandpass filters respectively. DAPI was excited with the violet laser diode (405 nm, 15 mW) and detected through a 461/50 nm bandpass filter. Samples were scanned in 0.5 μm steps and saved both as cytometric data and as PMT-reconstructed images. Data was acquired and analyzed using WinCyte software version 3.7.1 (Compucyte).

### Kinetic study of QD uptake using live cell microscopy

Fluorescent and Phase images of viable cells were acquired on an Axiovert 200 Cell Observer inverted microscope system from Zeiss (Oberkochen, Germany) that included an incubation enclosure around on the microscope stage. This system maintained normal cell culture conditions (37°C, 5% CO_2 _atmosphere, 100% relative humidity) and allowed multiple regions of interest to be imaged regularly (every 20 min in this study) throughout the duration of the experiment. Fluorescence from QD655 was detected through a 655/40 nm bandpass filter. Fluorescence images were processed digitally to correct for spatially uneven fluorescence excitation and for background fluorescence from QDs that remained suspended in the media solutions. Uneven fluorescence excitation was corrected by normalizing all images by a flat field image [19]. The flat field image was generated by imaging a spatially homogeneous 475 nm long pass glass filter. Correction for background fluorescence was simple background intensity subtraction where the fluorescence intensity attributed to background was determined from cell-free areas (as determined by phase contrast images) within each region of interest. The background fluorescence varied during the experiment, so the background fluorescence intensity was determined at each time point. The total intensity over the whole image was then summed to yield a measurement of the relative accumulation of QDs by cells within the region of interest.

### Intracellular localization of QDs

Cells grown on tissue culture chamber slides were treated with 0.8 nM QD655-COOH for 12 h. The culture medium was then removed and replaced with medium pre-warmed to 37°C containing dyes (final concentration 200 nM) for probing intracellular organelles including ER-Tracker Blue/White DPX for labeling endoplasmic reticulum (ER), MitoTracker Green for mitochondria, and LysoTracker Yellow for lysosomes, all obtained from Invitrogen. Cells were incubated with the dyes for 30 min, then replaced with fresh medium, followed by fixation and counterstaining with DAPI as described previously. Finally, the cells were observed under fluorescence microscope fitted with the correct filter set. Images were recorded separately in each fluorescence channel and merged afterwards.

### Transmission electron microscopy (TEM)

Cells were grown to confluence in culture flasks and treated with 0.8 nM QD655-COOH for 12 h at 37°C. Cells were then scraped into a centrifuge tube, washed 3 times with phosphate buffered saline (PBS), and fixed in a 2% glutaraldehyde solution diluted in 0.12 M Millonig's phosphate buffer (pH = 7.3). Whole mounts of primary fixed samples were washed in DI water, post-fixed with osmium tetroxide, dehydrated in sequential ethanol solutions, embedded in resin and finally ultramicrotombed. TEM images were obtained at 100 kV on a Zeiss EM10 CA electron microscope.

### Cytotoxicity assay

Cytotoxicity was measured by the MTS assay [20] using the CellTiter 96 Aqueous One Solution Cell Proliferation Assay kit from Promega (Madison, WI). Instructions from the manufacturer were followed. Briefly, cells were seeded in a 96-well plate at 1 × 10^4 ^cells/well and allowed to adhere overnight at 37°C with 5% CO_2_. Then cells were treated with QDs as described above and incubated for another 72 h. Afterwards the medium containing QDs was replaced with 100 μl fresh medium and 20 μl of assay reagent was added to each well. Cells were further cultured for 3 h and the resultant absorbance was recorded at 490 nm using a 96-well plate reader. Each experiment was performed with 3 independent replicates and repeated three times.

## Results

### Cellular uptake of QDs with different coatings

**Figure 1:**
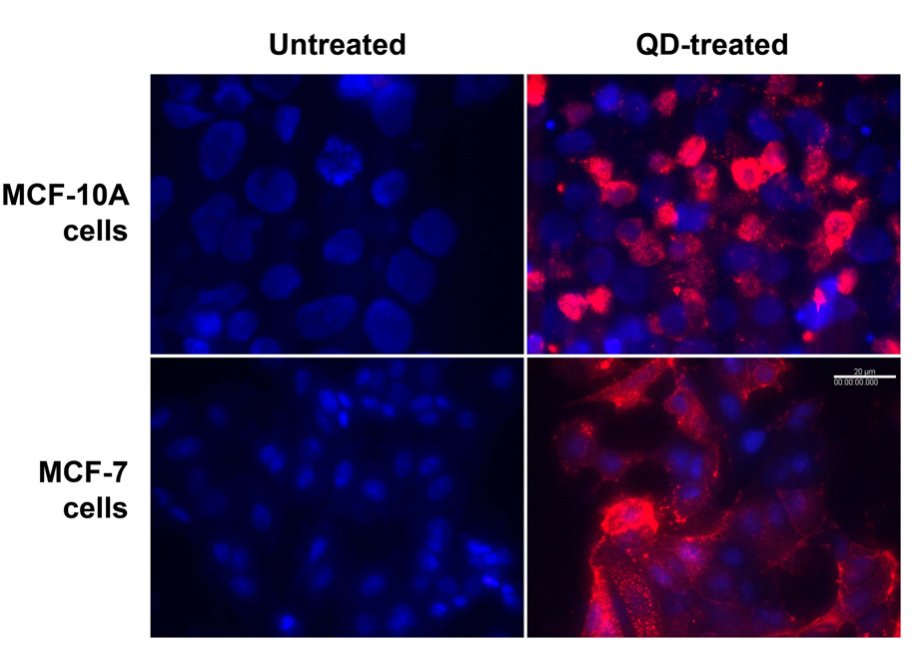
**Cellular uptake of QDs in human mammary non-tumorigenic MCF-10A cells and carcinoma MCF-7 cells**. The QD-treated cells were incubated with 0.8 nM QD655 coated with carboxylic acid (COOH) for 12 h at 37°C. Blue color represents DAPI-counterstained nucleus, while red color was fluorescence emitted from QD655. The white bar represents 20 μm.

Human mammary non-tumorigenic MCF-10A cells and carcinoma MCF-7 cells were incubated with QD655 of different coatings: carboxylic acid (COOH), amine-derivatized PEG or PEG only. No detectable intracellular uptake was observed for either amine-PEG or PEG coated QDs over 12 h incubation period (data not shown). However, both cell types internalized large amount of QD655-COOH after 12 h incubation (Figure 1). The internalized QDs formed large agglomerates localized around the periphery of the nuclei. It was observed that the percentage of cells taking up QDs is slightly higher in the cancerous MCF-7 cells than in the non-tumorigenic MCF-10A cells.

### Quantitation of QD uptake by laser scanning cytometry (LSC)

**Figure 2:**
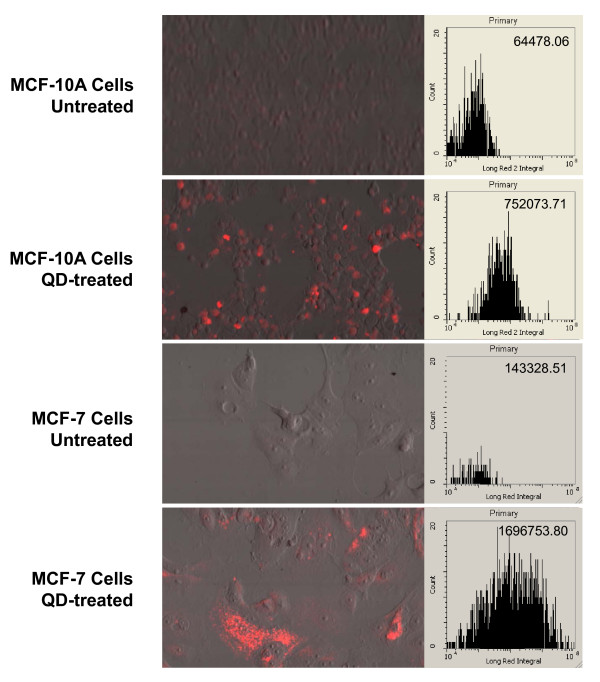
**Laser-scanning cytometry experiments quantitating QD uptake by MCF-10A and MCF-7 cells**. Representative PMT-reconstructed images are shown on the left panel. The cytometric data for peripheral fluorescence intensity (with spatial exclusion of the nucleus by DAPI contouring) collected from a channel optimized for QD655 (designated as Long Red 2) are shown on the right panel, with the average fluorescence intensity indicated at the top-right corner.

To quantitate QD uptake by MCF-10A and MCF-7 cells, we performed identical experiments using QD655-COOH and evaluated the results by laser-scanning cytometry. Representative PMT-reconstructed images are shown in Figure 2. Similar to Figure 1, high levels of QD fluorescence were detected inside the QD-treated cells for both cell types. The cytometric data for peripheral QD655 fluorescence intensity (with spatial exclusion of the nucleus by DAPI contouring) are shown in the left panel of the figure with the average fluorescence intensities indicated. The normalized average fluorescence intensity (average fluorescence intensity of QD-treated cells subtracted by that of the untreated cells) for MCF-7 cells (1,553,425.29) was ~2.3-fold as high as that for MCF-10A cells (687595.65). This result is concordant with the finding that higher percentage of MCF-7 cells internalized QD655-COOH than MCF-10A cells.

### Kinetic study of QD uptake

**Figure 3:**
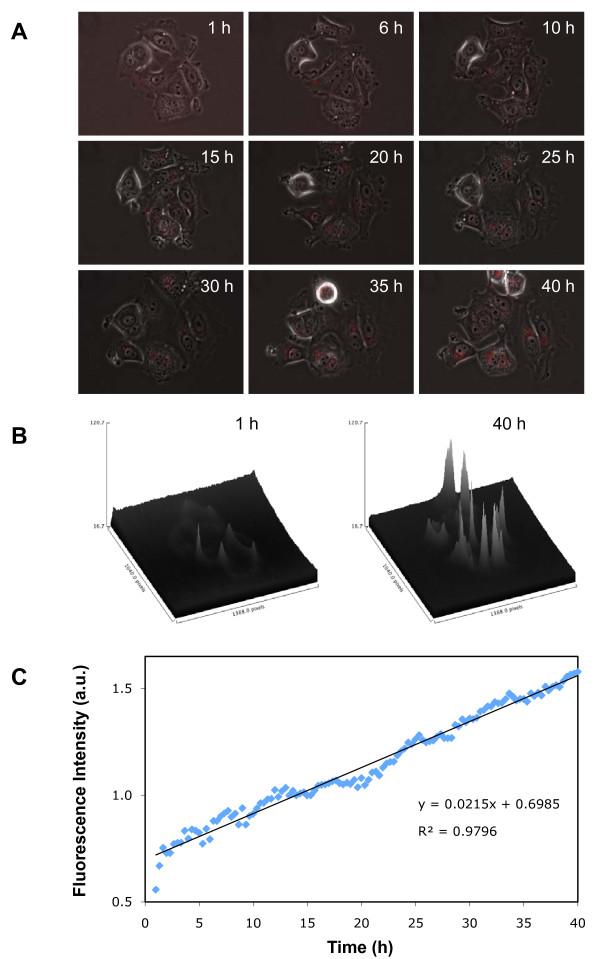
**Kinetics of QD655-COOH uptake by MCF-7 cells**. Images were taken every 20 min during 40 h of incubation using a live cell microscopy. **a**. Representative microscopic flat field images at specific time-points indicated. **b**. Three-dimensional graphs showing intracellular fluorescence intensity of the imaged area at 1 h and 40 h of incubation. The fluorescence intensity was corrected by subtracting background fluorescence as determined from cell-free areas of the region of interest. **c**. Plot of intracellular fluorescence intensity of the region of interest over time. Blue dots are fluorescence intensity at each timepoint; the straight line is linear regression.

The kinetics of QD655-COOH uptake by MCF-7 cells was studied using a live cell microscopy. Images were taken every 20 min during 40 h of incubation (Figure 3). QD fluorescence inside the cells became visible after ~1 h of incubation and increased almost linearly with time. The whole process can be visualized in a video clip provided as Additional file 1.

### Intracellular localization of QDs

**Figure 4:**
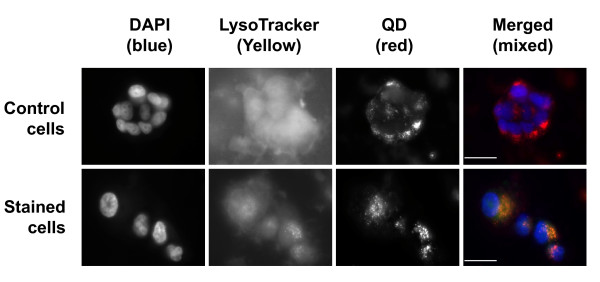
**Colocalization of QDs with lysosomes**. MCF-7 cells were treated with QD655-COOH for 12 h then incubated with LysoTracker Yellow for specific staining of lysosomes. Fluorescence from each channel was recorded and merged. The orange color seen in the stained cells resulted from the merging of the red fluorescence from QDs and the yellow color of the LysoTracker dye. The white bars represent 20 μm

To find out the intracellular localization of the internalized QDs, MCF-7 cells were treated with QD655-COOH for 12 h then incubated with dyes for probing intracellular organelles including ER, mitochondria, and lysosomes. Fluorescence microscope images showed that QDs colocalized with lysosomes (Figure 4) but not with ER or mitochondria (data not shown). This suggests that QDs were finally localized within the lysosomes.

### QD cellular uptake and intracellular translocation process

**Figure 5:**
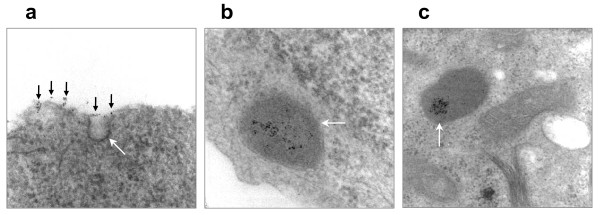
**TEM images illustrating the process of QD cellular uptake and intracellular translocation**. **a**. QD endocytosis through plasma membrane invagination. Black arrows point to QDs attached to the cell surface; the white arrow denotes the membrane pit engulfing QDs. **b**. QDs sequestered and dispersed in early endosomes (white arrow). **c**. QDs condensed in late endosomes/lysosomes (white arrow).

To shed light on the internalization of QDs by cells and their intracellular translocation process, MCF-7 cells were incubated with QD655-COOH and various stages of QD intracellular translocation were snapshot using TEM (Figure 5). QDs attached to the cell surface were engulfed through the formation of flask-shaped invaginations on the plasma membrane (Figure 5a). After pinching off the cell membrane, QDs were sequestered in the endocytic vesicles or early endosomes (Figure 5b), which slowly acidified and turned into late endosomes and lysosomes (Figure 5c). It is worth to note that QDs were dispersed in early endosomes (near neutral pH) but more densely packed in late endosomes/lysosomes, presumably due to the acidic pH therein.

### QD cytotoxicity on MCF-7 and MCF-10A cells

MCF-7 and MCF-10A cells were incubated with 0.8 nM QD655-COOH for 72 h and cell viability was examined by the MTS assay. No detectable decrease in cell viability was observed for both cell types (data not shown). Microscopic observations revealed that both cells appeared healthy after QD treatments without noticeable morphological changes.

## Discussion

**Figure 6:**
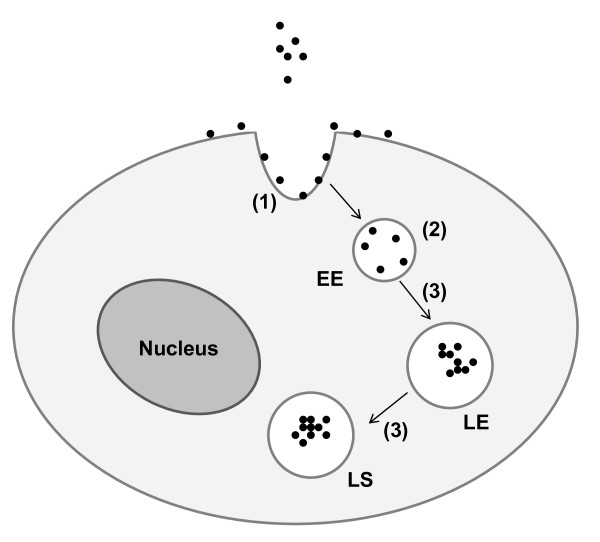
**Postulated QD cellular uptake pathway**. The process comprises of three major stages: (1) endocytosis; (2) sequestering in early endosomes (EE); (3) translocation to later endosomes (LE) or lysosomes (LS).

In summary, the results presented here suggest a potential pathway for QD cellular uptake mechanism, as illustrated in Figure 6, which comprises of three major stages: (1) endocytosis; (2) sequestering in early endosomes; (3) translocation to later endosomes or lysosomes (Figure 6).

Endocytosis of nanoparticles by cells may occur through two major mechanisms named phagocytosis and pinocytosis [21]. Phagocytosis is the uptake of large particles by only some specialized mammalian cells such as macrophages, monocytes, and neutrophils. Pinocytosis is for the uptake of small particles, solutes and fluid, and can be found in any cell type. Pinocytosis can be further classified into four subcategories: macropinocytosis, clathrin-mediated endocytosis, caveolae-mediated endocytosis, and clathrin/caveolae-independent endocytosis. Macropinocytosis, through cell surface ruffling, represents an efficient way for non-selective cellular uptake of large solute macromolecules with sizes >1 μm; while the other three, collectively called micropinocytosis, is preferred for the uptake of smaller particles through the formation of endocytic vesicles of different sizes - clathrin (~120 nm), caveolae (~60 nm) and clathrin/caveolae-independent (~90 nm) (see [22] for a detailed review). Based on the size of the QD655-COOH used in this study (hydrodynamic diameter 20-30 nm [15]), it is very likely that QD endocytosis by breast epithelial cells is mediated through micropinocytosis rather than macropinocytosis. It has been shown that macropinocytosis is not involved in QD uptake pathways in human epidermal keratinocytes (HEKs) [15].

Clathrin-mediated endocytosis is the most important mechanism for receptor-mediated uptake, occurs constitutively in all mammalian cells, and plays important physiological roles by carrying out the continuous uptake of essential nutrients such as the cholesterol-laden low-density lipoprotein (LDL) [23]. The endocytosis is mediated through the formation of clathrin-coated pits that are of 100-200 nm in size [24]. Several types of nanoparticles have been shown to enter cells through clathrin-mediated pinocytosis, such as FITC-labeled SPION and PEG-PLA [25,26]. Caveolae are flask-shaped plasma membrane invaginations of 50-80 nm size rich in cholesterol and sphingolipids, with shape and structural organization conferred by caveolin [27]. Caveolae-mediated endocytosis is most notably found in endothelial cells, smooth muscle cells and adipocytes. The physiological role of caveolae-mediated endocytosis may include cholesterol uptake, solute transport and tumor suppression [22,28]. Zhang and Monteiro-Riviere [15] reported that QD655-COOH internalization by HEK cells was via caveolae/lipid raft-mediated endocytosis involving LDL receptors (LDLRs) and scavenger receptors (SRs). This result is somewhat confusing and need to be further confirmed, as LDLRs are mainly associated with clathrin-mediated endocytosis [23]. In addition, SV40 virus entering cells via caveolae do not fuse with lysosomes after endocytosis [29]; however, QDs were localized in lysosomes in HEKs [15] and in mammary epithelial cells as shown in the current study. Based on these results, we hypothesize that QD655-COOH uptake by breast epithelial cells is most likely through clathrin-mediated endocytosis. Clathrin/caveolin-independent endocytosis has only been described in a few examples, *e.g*., for the recovery of membrane proteins in neurons or the internalization of the interleukin-2 (IL-2) receptor on lymphocytes [22]. The exact mechanism involved in cellular uptake of QDs may depend on many factors, such as the size and surface coating/charge of QDs, the type of cells, *etc*., more extensive studies are therefore needed to clarify this point.

In stark contrast to the rapid and large intracellular uptake of QD655-COOH, no detectable uptake was observed for either amine-PEG or PEG coated QDs. Similar findings have been reported for QD cellular uptake by HEK cells [15] and by murine macrophages [30]. The reason for this conspicuous difference is unknown, but most probably has to do with surface charge on the QDs. At physiological pH, the surface charge on QD655-COOH is negative, but is positive or neutral on amine-PEG or PEG coated QDs, respectively. The impact of surface charge on cellular uptake of non-targeted QDs has been studied sporadically and the results have been so far controversial; some studies reported negatively charged QDs can be internalized by cells [30-32], while others reported positively charged QDs can be endocytosed [33], still other studies showed surface coating/charge has no effect on QD endocytosis [34]. The exact mechanism is unknown, and may be cell type specific. However, it is very likely that the endocytosis involved in the internalization of QD655-COOH by the MCF-10A or MCF-7 cells was mediated by receptors that are specific or preferential to anionic ligands. Receptors favoring cationic ligands such as cell surface proteoglycans had been reported [35]. It has been suggested that LDLR/SR was involved in the internalization of QD655-COOH by HEK cells [15], since the size/charge of LDL or acetylated LDL (AcLDL), which are recognized by these receptors, are very similar to those of QD655-COOH.

However, other possible reasons for the preferential uptake of QD655-COOH could not be excluded. The QD655-COOH has an amphiphilic surface coating, while the other two QD types contain a PEG-based outer coating on top of the amphiphilic inner coating [36]. Thus, the surface of QD655-COOH could be more hydrophobic than that of QDs coated with amine-PEG or PEG. The higher hydrophobicity for QD655-COOH may facilitate the transport of the QDs through the cell membrane. However, further studies are needed to clarify the mechanisms for the differential cellular uptake of the QDs.

The observed condensation of QDs upon translocation from early endosomes to late endosomes/lysosomes was probably a result of the pH change in these endocytic compartments. The pH value in early endosomes is 5.9-6.0 [37] therefore the QD655-COOH particles are negatively charged and expels one another and stay dispersed. In lysosomes, the pH drops to 5.0-5.5 [34] and in some cases can be as low as 4 [37,38], at which the carboxyl groups on the QD surface strongly protonate and become practically neutral, thus resulting in QD aggregation. The protonation of QD surface may result in an increase of intraendosomal pH and a charge gradient provoking a water influx and endosomal swelling and disintegration, resulting in the escape of QDs from the endo-lysosomal compartment [13]. This phenomenon could be utilized to target drug-laden QDs to the cytoplasm [39].

MCF-7 is a mammary carcinoma cell line while MCF-10A is a non-tumorigenic cell line. Both cell types internalized large amount of QD655-COOH, although the percentage of endocytosing cells is slightly higher in MCF-7 cells than in MCF-10A cells. This result implies that both normal and cancerous cells are able to passively internalize significant amount of QDs without conjugation with specific targeting moieties. Therefore, targeting QDs specifically to cancer cells would not be achievable unless passive QD delivery is blocked or minimized. A well known solution is to mask the surface of QDs with PEG, which can significantly reduce non-specific cellular uptake of nanoparticles [40]. It has been shown that surface modification with PEG remarkably reduced non-specific QD uptake by many cell types [41,42]. The results presented in this study that QDs coated with PEG or amine-derivatized PEG were not internalized by the cells add further evidence to the effectiveness of this method. An importance inference from these results is that future applications for specific targeting of cancer cells should use QDs coated with PEG or PEG derivatives.

One major obstacle to clinical applications of QDs is the concern over their possible cytotoxicity [7]. Cd^2+ ^ions can be released through oxidative degradation of QDs, and then bind to thiol groups on intracellular proteins. Also, QDs may aggregate, precipitate on cells, non-specifically adsorb to biomolecules, and catalyze the formation of reactive oxygen species (ROS), all of which contribute to QD toxicity. In addition, little is known about the degradation, metabolism and body clearance of QDs. The unique structure of QDs presents a complex set of physic-chemical parameters that confounds systematic studies on toxicity mechanisms of QDs, such as composition, size, surface coating, and bioconjugation, *etc*. Like most studies in the past, the toxicity study reported here is primarily observational in nature. Although the results indicated that no cytotoxic effects of QDs were observed over an incubation period of 72 h, the large amount of QDs accumulated inside the cell and their persistence in the lysosomes underscore the need for long-term studies of QD toxicity and fate in cells and clearly emphasizes the importance of strict environmental control of QDs and other nanoparticles as well.

## Conclusions

Surface coating has a profound impact on the cellular uptake of QDs. PEG modification essentially blocked non-specific QD delivery into the cells. On the other hand, QDs coated with COOH were internalized quickly and with large amount by both cancerous and non-cancerous cells. QD cellular uptake involves three major stages including endocytosis, sequestration in early endosomes, and translocation to later endosomes or lysosomes. The endocytosis was probably assisted by receptors specific to ligands with negative charges. These findings could be exploited to reduce non-specific targeting, thereby improving specific targeting of QDs in cancer diagnosis and treatment applications. The findings are also important in understanding the cytotoxicity of QDs and other nanomaterials in general and in emphasizing the importance of strict environmental control of nanoparticles.

## Competing interests

The authors declare that they have no competing interests.

## Authors' contributions

YX conceived of the study, designed and carried out most of the experimental work, coordinated the project, analyzed the data, and drafted the manuscript. SPF carried out the kinetic study using live cell microscopy, and analyzed the data. XG participated in the design of the study, performed data analysis, and drafted the manuscript. RDH carried out the TEM studies. WGT participated in the laser scanning cytometry study and analyzed the data. AT carried out cell culture for the studies. All authors read and approved the final manuscript.

## Supplementary Material

Additional file 1 **Real-time live cell imaging of QD cellular uptake**. Click here for file
